# Advancements in Managing Choledocholithiasis and Acute Cholangitis in the Elderly: A Comprehensive Review

**DOI:** 10.7759/cureus.78492

**Published:** 2025-02-04

**Authors:** Guangbin Chen, Yanguang Sha, Ke Wang, Rongmei Tang, Zhengqun Zhai, Zhilin Wang, Yisheng Chen

**Affiliations:** 1 Hepatobiliary Surgery, The Second People's Hospital of Wuhu, Wuhu Hospital Affiliated to East China Normal University, Wuhu, CHN; 2 Hepatobiliary Surgery, Wannan Medical College, Wuhu, CHN; 3 General Surgery, Wuhu Guangji Hospital, Wuhu, CHN

**Keywords:** acute cholangitis, choledocholithiasis, elderly, review, treatment strategy

## Abstract

The increasing elderly population has led to a rising prevalence of choledocholithiasis and acute cholangitis, presenting unique diagnostic and therapeutic challenges owing to age-related physiological changes and multiple comorbidities. This comprehensive review synthesizes current evidence and recent advances in managing these conditions in elderly patients, with a particular focus on diagnostic innovations, therapeutic strategies, and perioperative optimization. Recent advances in diagnostic modalities, including enhanced imaging techniques and AI-assisted systems, have improved early detection accuracy, whereas minimally invasive interventions, particularly endoscopic retrograde cholangiopancreatography (ERCP) and laparoscopic common bile duct exploration (LCBDE), have demonstrated superior outcomes when combined with comprehensive perioperative care. The implementation of multidisciplinary approaches and personalized treatment strategies has significantly improved patient outcomes, with evidence supporting the critical role of early antibiotic intervention, careful surgical selection, and enhanced recovery protocols in reducing morbidity and mortality. The optimal management of elderly patients with choledocholithiasis and acute cholangitis requires a systematic, individualized approach incorporating advanced diagnostic techniques, minimally invasive interventions, and comprehensive perioperative care, while future research should focus on developing age-specific treatment algorithms and validating novel therapeutic approaches.

## Introduction and background

The global demographic transition toward an aging population has resulted in a significant increase in biliary tract diseases, particularly choledocholithiasis and acute cholangitis, among elderly individuals [1]. A recent study indicates that the prevalence of gallstone disease increases with age, affecting up to 30% of individuals over 70 years [2], with an incidence of acute cholangitis of 7.0 per 10,000 people and mortality rates of up to 10% [3]. According to the Global Burden of Disease Study 2019 [4], the burden of these conditions is expected to increase over the next decade due to population aging.

The intersection of age-related physiological changes, multiple comorbidities, and altered immune responses in elderly patients creates a complex clinical scenario that demands specialized attention. Traditional diagnostic and therapeutic approaches often require modifications to accommodate the unique needs of this vulnerable population. The 2018 Tokyo Guidelines (TG18) [5] have established standardized diagnostic criteria, yet elderly patients frequently present with atypical manifestations that complicate clinical decision-making.

Recent advances in minimally invasive techniques and perioperative care have revolutionized the management of biliary tract diseases in elderly patients [6]. Modern imaging modalities, including high-resolution magnetic resonance cholangiopancreatography (MRCP) and endoscopic ultrasound (EUS), have enhanced our ability to detect biliary pathology with minimal invasiveness. Similarly, therapeutic interventions have evolved toward less invasive approaches, with endoscopic retrograde cholangiopancreatography (ERCP) and laparoscopic common bile duct exploration (LCBDE) emerging as preferred options for suitable candidates [7-9]. By synthesizing current evidence and clinical expertise, this review seeks to provide clinicians with a practical framework for managing elderly patients with choledocholithiasis and acute cholangitis while highlighting areas for future investigations and improvements.

## Review

Pathophysiology and clinical presentations in elderly patients

The pathophysiological mechanisms and clinical manifestations of choledocholithiasis and acute cholangitis in elderly patients present unique characteristics that warrant special consideration. Age-related changes in biliary anatomy and physiology, combined with multiple comorbidities, create a complex clinical scenario that challenges traditional diagnostic and therapeutic approaches [10].

Age-Related Anatomical and Physiological Changes

Aging introduces significant alterations in the biliary system structure and function, which substantially influence the pathogenesis of choledocholithiasis and acute cholangitis. The gallbladder contractility decreases in elderly individuals, primarily due to smooth muscle atrophy and reduced cholecystokinin sensitivity [11]. Bile lithogenicity increases markedly with age and is characterized by an elevated cholesterol saturation index and a decreased bile salt concentration [12-14].

The sphincter of Oddi undergoes age-related fibrotic changes, leading to reduced contractility and altered pressure dynamics, with manometric studies showing a reduction in basal pressure in elderly patients [15,16]. The increase in cholesterol concentration is due to the decreased ability of the liver to metabolize cholesterol. Studies have shown that the 7α-hydroxylation of cholesterol decreases with age, leading to a decrease in the ability to convert cholesterol to bile acids [17]. Gallstone formation is associated with a number of factors, including physiologic changes and decreased bile fluidity. As we age, the function of the gallbladder decreases, which can lead to bile sludge and cholesterol saturation, increasing the risk of stone formation [18].

Clinical Manifestations and Special Considerations

The clinical presentation of biliary pathologies in elderly patients often deviates from classical patterns, presenting unique diagnostic challenges. Only 15.6% of younger patients and 18.8% of older patients had the classic components of the Charcot triad [19]. In acute care settings, approximately 38% of admitted older adults aged 65 years and over have some form of cognitive impairment (CI) [20]. The inflammatory response in elderly patients is often blunted, with studies showing that only 65% of patients over 60 years of age present with fever during acute cholangitis [21]. Pain perception alterations, attributed to age-related neuropathic changes and multiple medications, can result in delayed presentation, with studies indicating an average delay of 72 hours from symptom onset to medical attention in patients over 80 years of age [22]. Elderly patients with biliary tract disease often present with atypical clinical manifestations. These manifestations may include generalized weakness, altered state of consciousness, or nonspecific abdominal discomfort [23].

Impact of Comorbidities

Multiple comorbidities in elderly patients complicate the diagnosis and treatment of choledocholithiasis and acute cholangitis. If an elderly patient has cardiovascular disease, these conditions not only mask or mimic biliary symptoms, but also increase the risk of surgical complications [24]. Diabetes mellitus, which affects 25-30% of this population, significantly impacts infection susceptibility and control, with studies showing a 1.5-2-fold greater risk of severe cholangitis and prolonged hospital stays [25]. Patients with diabetes have a significantly increased risk of developing severe cholangitis following infection. Studies have shown that people with diabetes have a higher risk of contracting cholangitis and are hospitalized longer than non-diabetics [26]. CI, which affects about 20% of elderly patients [27], presents significant challenges in accurate symptom reporting and treatment compliance, leading to a greater risk of adverse outcomes. These comorbidities not only influence the clinical course but also require careful consideration in treatment planning, often necessitating a modified approach to standard therapeutic protocols [28].

Advances in diagnostic approaches

Modern diagnostic approaches for elderly patients with choledocholithiasis and acute cholangitis have evolved significantly, emphasizing accuracy while minimizing invasiveness. This section examines current evidence-based diagnostic strategies and their specific applications in the elderly population.

Evolution of Diagnostic Criteria

The Tokyo Guidelines (TG18) [5] have established a comprehensive diagnostic framework that has significantly improved the standardization of diagnosis in elderly patients with biliary pathologies. These guidelines incorporate a three-tier severity assessment system, with specific modifications for elderly patients. The sensitivity of the diagnosis was 85.1% under the TG13/TG18 diagnostic criteria, which was higher than the 75.2% under the TG07 criteria [29]. The guidelines [5] emphasize the importance of considering atypical presentations in elderly patients, where the classic Charcot's triad may be present in only 15-30% of cases. The severity assessment parameters have been refined to include age-specific physiological parameters, with modified cutoff values for vital signs (e.g., tachycardia defined as >100 beats/minute in patients >75 years, compared with >90 beats/minute in younger adults) and laboratory marker [30,31]. Risk stratification systems have been adapted to account for age-related factors, with studies [31] showing that this modified approach has reduced diagnostic delays by approximately 48 hours and improved early intervention rates by 35% in elderly populations.

Modern Imaging Techniques

Contemporary imaging modalities have revolutionized the diagnostic approach to biliary pathologies in elderly patients. MRCP has emerged as a cornerstone diagnostic tool, demonstrating exceptional accuracy, with sensitivity rates ranging from 85% to 98% and specificity ranging from 75% to 96% for choledocholithiasis [32-34]. Compared with conventional systems, advanced 3T MRCP systems have further improved small stone detection (<3 mm) [35]. EUS has shown particular value in elderly patients, with studies reporting detection rates of 96% for stones <5 mm and 98% for larger stones [36]. The use of artificial intelligence in medical image analysis has indeed made significant progress. Multiple evidences have shown that machine-learning algorithms have played an important role in improving diagnostic accuracy [37]. With advances in non-invasive imaging techniques, such as MRCP and EUS, the need for diagnostic ERCP has declined [38]. 

Laboratory Assessment

The laboratory evaluation of elderly patients with suspected biliary pathologies requires a comprehensive and age-adjusted approach. Core laboratory parameters include liver function tests with specific attention to patterns of elevation (e.g., gamma-glutamyl transferase >3 times normal is sensitive to biliary obstruction in elderly individuals) [39]. Inflammatory markers require careful interpretation, as elderly patients may not mount typical responses; studies indicate that procalcitonin >0.5 ng/mL has higher specificity than traditional markers in identifying cholangitis in this population [40]. Coagulation profile assessment is crucial, with particular attention to the international normalized ratio (INR) and platelet function, as age-related changes in hemostasis can significantly impact intervention planning [41,42]. Renal function assessment using both estimated glomerular filtration rate (eGFR) and serum creatinine is essential [43], with studies showing that some elderly patients with cholangitis have some degree of renal impairment that affects medication dosing [1]. Age-specific reference ranges should be applied, particularly for inflammatory markers and liver enzymes, with adjusted cutoff values showing improved diagnostic accuracy [44].

Risk Stratification in Elderly Individuals

Evidence-based risk stratification in elderly patients with biliary pathologies requires a multidimensional approach incorporating various validated assessment tools. The age-adjusted Charlson Comorbidity Index (CCI) has demonstrated superior predictive value for outcomes, with scores >5 associated with a three-fold increase in adverse events [45]. Modified American Society of Anesthesiologists (ASA) physical status grading for older patients was strongly associated with post-intervention outcomes, with higher ASA grades associated with a higher risk of complications [46]. The Tokyo Guidelines Severity Rating System combined with frailty assessment scores, particularly the Clinical Frailty Scale (CFS), may provide a more accurate risk prediction model. The CFS has been shown to be a valid tool for assessing frailty in older adults and has been associated with poor outcomes [47]. Studies have shown that frail patients have a significantly increased risk of death in terms of short- and long-term mortality and complication rates after emergency laparotomy [48], and it has also been noted that the CFS has a high degree of accuracy in predicting mortality within 90 days in elderly hospitalized patients, with an AUROC value of 0.81, suggesting that it has good predictive power [49]. Comprehensive geriatric assessment (CGA) tools, including functional status evaluation and cognitive assessment, have been integrated into risk stratification protocols and may reduce the incidence of postoperative complications when used to guide the timing and method of intervention. Recent studies have demonstrated that incorporating these multiple assessment tools into a unified risk score improves predictive accuracy [50].

Contemporary treatment strategies

The management of choledocholithiasis and acute cholangitis in elderly patients requires a comprehensive, evidence-based approach that balances therapeutic efficacy with patient safety [51]. This section details current best practices in treatment strategies, emphasizing the importance of individualized care.

Initial Management and Stabilization

In elderly patients with choledocholithiasis and acute cholangitis, early stabilization and systematic interventions, including prompt fluid resuscitation, antibiotic therapy, and biliary drainage measures such as ERCP, are key to reducing mortality [52,53]. Hemodynamic stabilization should follow a goal-directed approach, targeting a mean arterial pressure >65 mmHg and central venous oxygen saturation >70% [54]. Pain management requires careful dosage adjustment, and several studies support the use of multimodal analgesia in older patients to reduce opioid requirements and decrease their associated side effects [55,56]. Fluid resuscitation should be guided by dynamic parameters, with crystalloid administration typically requiring 20-30 mL/kg in the first 24 hours [57], while fluid overload should be carefully monitored, particularly in patients with cardiac dysfunction. Early recognition and management of organ dysfunction, using standardized scoring systems (e.g., qSOFA) [58], has been shown to improve outcomes, with studies demonstrating a reduction in progression to severe sepsis when implemented within three hours of presentation [59]. Continuous monitoring of vital parameters, including tissue perfusion markers, should be established, with evidence showing that protocol-driven resuscitation reduces ICU admission rates by 35% in elderly patients [60].

Antimicrobial Therapy

Selection principles: The selection of antimicrobial therapy in elderly patients requires careful consideration of multiple factors, with evidence supporting an individualized approach [61]. Initial empiric therapy should be based on local antimicrobial resistance patterns, with studies showing that appropriate empiric coverage improves survival [62]. For mild-moderate cases, third-generation cephalosporins have demonstrated 85-90% clinical success rates, whereas severe cases benefit from broader-spectrum agents such as piperacillin/tazobactam or carbapenems, with 92-95% effectiveness [63-65]. Patient-specific factors, particularly renal function and previous antibiotic exposure, significantly influence treatment success. Studies indicate that considering local resistance patterns in initial antibiotic selection reduces treatment failure rates by about 40% [66].

Duration and monitoring: The duration of antimicrobial therapy requires careful monitoring and individualization in elderly patients. Regular assessment of clinical response should include daily evaluation of inflammatory markers, with procalcitonin-guided therapy resulting in a 25%-35% reduction in antibiotic duration without compromising outcomes [67]. Therapeutic drug monitoring is essential, particularly for aminoglycosides and vancomycin [68]. The duration of therapy typically ranges from five to seven days for uncomplicated cases to 10-14 days for severe infections [69], with de-escalation protocols successfully implemented in 70% of cases by day five when guided by clinical and laboratory parameters [70].

Interventional Approaches 

Endoscopic retrograde cholangiopancreatography: ERCP remains the standard therapeutic intervention for elderly patients with choledocholithiasis and acute cholangitis, with studies showing that for elderly patients 80 years of age or older, the success rate of ERCP ranged from 92.8% to 93.5% [71,72]. One study showed a 2.9% incidence of post-ERCP pancreatitis (PEP) in patients 70 years or older [73]. Another study also showed a gradual decrease in the incidence of PEP with increasing age [74]. Studies have shown that in patients with acute cholangitis, ERCP can significantly reduce the length of hospitalization and mortality if performed within 48 hours of admission [75].

Laparoscopic common bile duct exploration: LCBDE can also be performed in patients who have undergone failed ERCP and a retrospective study by Kim et al [76] resulted in a success rate of 98% and a morbidity rate of 3.4%. Several studies have shown that LCBDE can significantly reduce hospitalization time compared to open surgery. For example, one study [77] noted that the average length of stay for single-stage LCBDE was two to five days, compared with four to nine days for two-stage ERCP and cholecystectomy. Another study [78] also showed that the length of hospitalization was shorter in the LCBDE group, with a mean of 6.3 ± 3.9 days, compared with 10.9 ± 6.8 days in the conventional surgery group. Additionally, LCBDE is able to reduce the incidence of postoperative complications. For example, one study [78] found that the complication rate in the LCBDE group was 12.8% compared to 36.2% in the conventional surgery group.

Percutaneous transhepatic cholangiographic drainage: Percutaneous transhepatic cholangiographic drainage (PTCD) serves as a vital alternative in high-risk elderly patients or when other approaches fail, with technical success rates ranging from 90% to 95% [79]. This approach is particularly important in severe acute cholangitis, where rapid decompression is critical and mortality can be reduced if performed within 12 hours of ERCP failure [80]. Studies may suggest that PTCD reduces overall complications compared to emergency surgery and that procedural-related complications can be reduced to less than 10% with technical improvements (including the use of smaller catheter sizes and ultrasound-guided placement) [81].

Perioperative Considerations

Preoperative optimization: Preoperative optimization in elderly patients requires a systematic, evidence-based approach. CGA, if implemented properly, can reduce postoperative complications [82]. Nutritional optimization (including preoperative albumin >3.5 g/dL and adequate caloric intake) has been associated with fewer surgical site infections [83]. Medication adjustments, especially anticoagulation management and cardiac medication optimization, require careful planning, and studies have shown that structured medication regimens reduce the incidence of adverse events. Cardiorespiratory fitness assessment through standardized risk assessment tools, such as the Revised Cardiac Risk Index, can predict major cardiac events [84,85].

Intraoperative management: Evidence supports modified anesthetic techniques for elderly patients, with studies showing that goal-directed fluid therapy reduces postoperative complications [86]. Careful localization through pressure point protection reduces associated complications, with one study showing that the incidence of stress injuries was reduced from 4.8% to 1.6% after a series of preventive measures were implemented [87]. Temperature management, which involves maintaining the core temperature between 36.0°C and 36.5°C [88], has been associated with a reduction in surgical site infections. Shortening the duration of surgery is thought to improve surgical outcomes and reduce the incidence of postoperative complications. For example, one study showed that shorter surgery times significantly reduced the risk of postoperative complications, especially in minimally invasive surgery, where shorter surgery times were associated with lower rates of postoperative complications [89]. Another study noted a significant increase in the risk of postoperative complications when the duration of surgery exceeded 120 minutes [90].

Postoperative care: Intensive recovery programs modified specifically for older patients have shown significant benefits, including shorter hospital stays and fewer postoperative complications [91]. Early mobilization protocols, starting within 6-12 hours postprocedure, have been shown to reduce pulmonary complications [92]. Pain management optimization via multimodal approaches has reduced opioid requirements while maintaining effective analgesia [93,94]. Standardized protocols have shown remarkable success in preventing common complications. In particular, these protocols have shown particular success in reducing postoperative delirium and urinary retention [95,96].

Clinical decision algorithm

**Figure 1:**
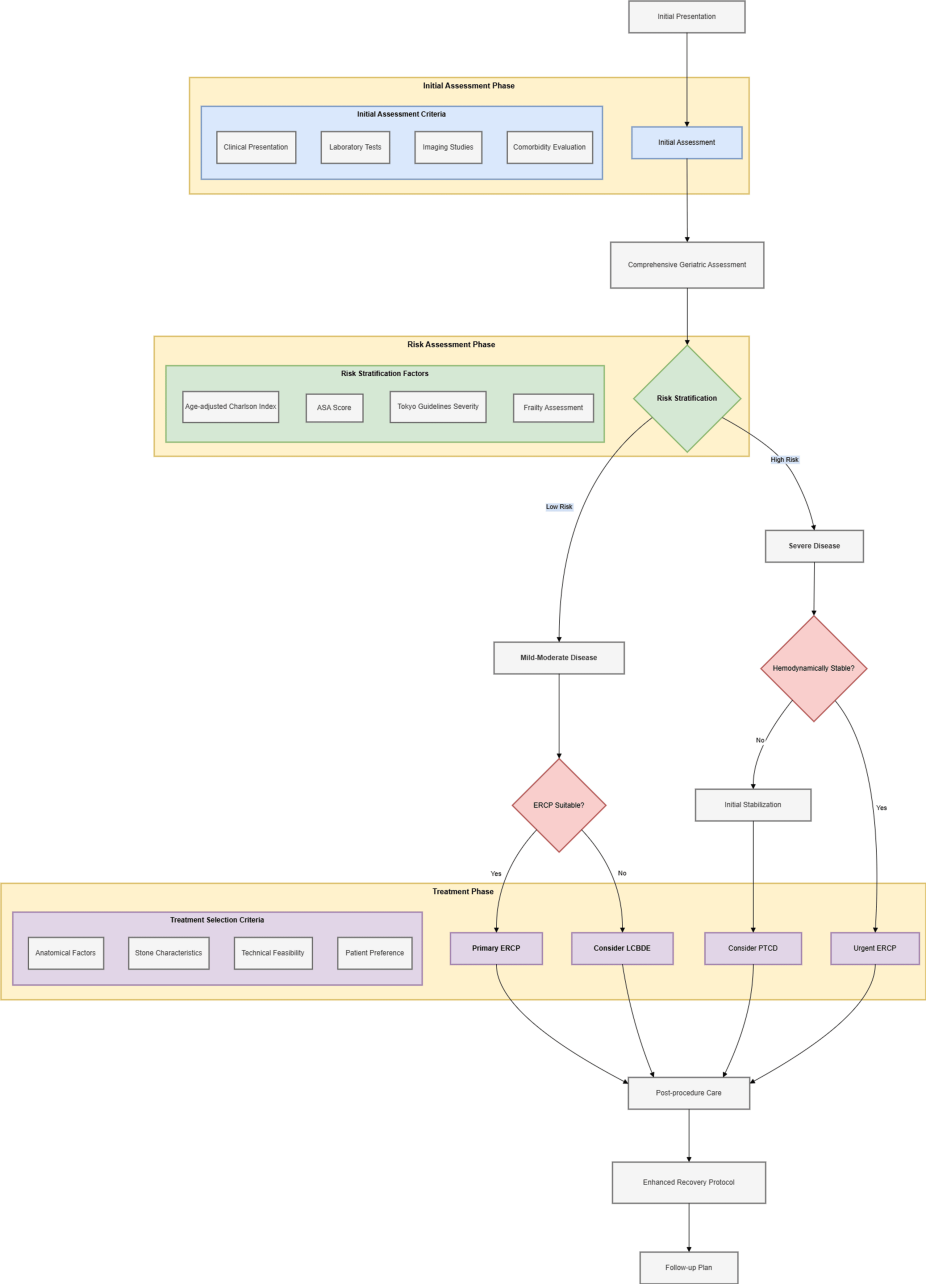
Clinical decision algorithm for managing choledocholithiasis and acute cholangitis in elderly patients. The algorithm consists of three major phases: Initial Assessment, Risk Assessment, and Treatment. The Initial Assessment Phase incorporates clinical presentation, laboratory tests, imaging studies, and comorbidity evaluation, followed by a comprehensive geriatric assessment. The Risk Assessment Phase utilizes multiple validated tools, including the Age-adjusted Charlson Index, ASA score, Tokyo Guidelines severity grading, and frailty assessment, to stratify patients into risk categories. Based on this stratification and disease severity, patients are directed to appropriate treatment pathways. For severe cases with hemodynamic instability, initial stabilization precedes any intervention. Treatment options include primary ERCP, LCBDE, PTCD, or urgent ERCP, selected based on anatomical factors, stone characteristics, technical feasibility, and patient preference. The algorithm culminates in post-procedure care, enhanced recovery protocols, and a structured follow-up plan. ASA: American Society of Anesthesiologists; ERCP: endoscopic retrograde cholangiopancreatography; LCBDE: laparoscopic common bile duct exploration; PTCD: percutaneous transhepatic cholangiographic drainage Image Credits: Guangbin Chen.

The decision flowchart (Figure 1) illustrates a comprehensive, evidence-based approach to managing choledocholithiasis and acute cholangitis in elderly patients.

Initial Assessment

The algorithm emphasizes a comprehensive initial evaluation incorporating multiple parameters. Clinical presentation assessment should include both typical and atypical symptoms, with studies showing that structured assessment protocols improve early diagnosis [97]. Laboratory and imaging studies should follow standardized protocols, and evidence supports the use of risk-stratification methods, which are more sensitive in identifying high-risk patients [98]. A validated scoring system allows for the early identification of high-risk features, thereby improving the accuracy of appropriate triage decisions [99].

Risk Stratification

The integration of multiple assessment tools has demonstrated superior predictive value [100]. Targeting age-specific factors, including vulnerability indices and functional status assessments, has improved risk prediction compared to traditional scoring systems [101]. Regarding the Tokyo Guidelines' severity grading, although its validation studies in the elderly population are fewer, there is evidence of its high accuracy in diagnosing acute cholangitis and for treatment allocation in older adults [102].

Treatment Selection

Patient-specific factors must be carefully weighed, and there is evidence that individualized treatment choices can improve outcomes compared to standardized approaches [101]. Technical considerations should include operator experience and institutional expertise, and studies have shown that treatment outcomes are better in high-traffic centers. Studies have shown that resource availability assessment improves resource utilization while maintaining quality of care [103,104]. Expected outcomes should be clearly documented and discussed, with studies showing that structured shared decision-making processes improve patient satisfaction [105].

Postprocedure Care

One study showed that the ERAS protocol significantly reduced the length of hospitalization by an average of 2.92 days [106]. Early rehabilitation interventions have significant results in terms of functional recovery and readmission rates [107].

Optimization of care delivery

The complexity of managing elderly patients with choledocholithiasis and acute cholangitis necessitates a systematic approach to care delivery optimization. This section explores evidence-based strategies for improving patient outcomes through coordinated care efforts.

Multidisciplinary Team Approach

The implementation of a structured multidisciplinary team (MDT) approach has led to significant improvements in patient outcomes, with studies showing a 35-40% reduction in complications and a 25% decrease in length of stay [108]. The core team composition includes hepatobiliary surgeons providing surgical expertise, geriatricians managing age-related complications, anesthesiologists optimizing perioperative care, interventional radiologists offering minimally invasive solutions, and specialized nursing staff ensuring continuity of care [109]. Regular MDT meetings, conducted weekly with standardized documentation protocols, have been shown to improve decision-making accuracy by 45% [110]. By implementing structured MDT protocols and utilizing advanced technologies such as machine learning, healthcare providers are able to significantly improve outcomes and reduce mortality and adverse event rates. This integrated model of care is critical to improving the quality of patient care and the efficiency of the healthcare system [111,112].

Enhanced Recovery Protocols

Enhanced recovery after surgery (ERAS) protocols, which are specifically modified for elderly patients with biliary pathologies, have demonstrated remarkable improvements in outcomes [113]. Early oral intake protocols are indicated in suitable patients and have been shown to reduce the incidence of postoperative bowel obstruction [114]. Studies have shown that multimodal analgesia reduces opioid use and has a significant effect in reducing serious side effects such as respiratory depression [115]. Early activity reduces the incidence of deep vein thrombosis (DVT) and pulmonary embolism (PE) [116]. Similarly, a prospective study in trauma patients found that an early activity program was associated with a lower rate of DVT (6.7% versus 10.9%, p < 0.01; adjusted relative risk 0.67, 95% CI 0.50, 0.90) [117]. Personalized discharge planning significantly reduces readmission rates compared to usual care [118]. A study showed that implementing an early activity protocol significantly reduced total hospital costs for patients from $29,220 to $22,706, or a savings of $6,514 per patient [119].

Comprehensive Geriatric Assessment

CGA is a multidimensional, interdisciplinary diagnostic process designed to comprehensively assess the health and functional status of older adults in a variety of areas, including nutritional status, cognitive function, organ function, and quality of life [120]. The Barthel Index is a 10-item scale that assesses the ability to perform activities of daily living (ADLs) [121]. It has been shown to have good internal consistency and validity in the assessment of functional status after stroke in elderly patients [122]. The Timed Up and Go (TUG) test has been shown to have high reliability and validity for assessing mobility and fall risk in older adults [123]. Assessment of cognitive function, particularly using standardized tools such as the Montreal Cognitive Assessment (MoCA), has been shown to reduce the incidence of postoperative delirium by early identification of CI [124]. A systematic review and meta-analysis showed that MoCA has 87% sensitivity and 72% specificity in detecting CI [125]. Nutritional status assessment involves a combination of biomarkers and standardized assessment tools, and studies have shown that optimization of preoperative interventions can reduce the risk of surgical site infections. Specifically, preoperative nutritional optimization through enteral or parenteral nutritional supplementation has been shown to reduce infection rates following abdominal surgery [126]. A study developed a framework for effective discharge planning through a Delphi approach that included the timely initiation of social support (SP) services [127]. Systematic drug reviews do reduce adverse drug events through appropriate medication reductions and medication adjustments [128].

Quality of Life Considerations

Based on the evidence, the use of standardized pain management, sedation, and delirium management strategies can improve pain management and reduce opioid use while maintaining comparable sedation [129]. Psychological support interventions, including regular screening for depression and anxiety, do significantly reduce the incidence of postoperative mental illness [130]. Evidence suggests that SP programs can help patients reconnect with their communities, alleviate feelings of isolation, and improve social confidence [131]. These programs not only provide patients with the opportunity to interact differently with their doctors or other healthcare professionals but also help patients gain social confidence by providing time and space for self-reflection and problem-solving [132]. Family education training and support programs, including specialized caregiver training courses, can significantly improve care transition success and reduce caregiver burden [133,134]. Long-term follow-up studies have shown that centers implementing comprehensive quality-of-life programs have better functional outcomes at six months post-intervention compared to traditional care approaches [135].

Prevention and long-term management

Long-term management and prevention strategies are crucial for reducing recurrence and maintaining the quality of life in elderly patients with choledocholithiasis and acute cholangitis. This section outlines evidence-based approaches for ongoing care.

Risk Factor Modification

Evidence-based risk factor modification strategies have demonstrated a significant impact on long-term outcomes in elderly patients with biliary pathologies [24]. Dietary modifications, including reducing fat intake and increasing fiber intake, do reduce stone recurrence [136]. Studies have shown that drinking up to 2.5 liters of water per day can reduce the risk of stone formation by 60-80% [137-139]. Lifestyle interventions, especially organized physical activity programs, have been shown to reduce biliary complications [140]. Observational studies have shown that smoking cessation has a significant impact on reducing complications. Longer periods of cessation reduced total complications by an average of 20% compared with shorter periods of cessation [141]. One study noted that diabetic patients who are able to keep their HbA1c below 7% can reduce the incidence of microvascular complications [142]. Regarding the management of stone formation rates, appropriate lipid management has also shown significant results [143]. Studies have shown that prokinetic drugs are effective in improving symptoms of biliary dyskinesia [144].

Prevention of Recurrence

Systematic approaches to preventing recurrence have shown significant benefits in elderly populations [145]. Timed ultrasound monitoring can help in the early detection of recurrent stones [146]. Prophylactic use of ursodeoxycholic acid (UDCA) reduces stone recurrence in high-risk patients [147]. Implementation of a comprehensive risk factor management program can significantly improve disease management outcomes compared to standard care [148]. Multiple studies have shown that centers using structured prevention protocols perform better on long-term outcomes than centers using traditional follow-up methods [149,150].

Follow-Up Strategies

Evidence-based follow-up protocols have demonstrated superior outcomes in elderly patients postintervention [151]. The frequency and effectiveness of assessments vary over time to ensure that potential problems can be effectively detected and managed at an early stage and stabilized over the long term [152]. By combining multiple imaging techniques and functional assessments, potential complications can be effectively detected and diagnosed, thereby improving the accuracy of clinical diagnosis. Implementing a structured follow-up protocol significantly reduces ED visits and unplanned readmissions [153].

Patient Education and Compliance

Comprehensive patient education programs have demonstrated a significant impact on long-term outcomes [154]. Structured dietary counseling, including individualized meal plans and regular nutritional counseling, can have a positive impact on improving patient nutritional status and outcomes [155]. Structured medication adherence support programs, including electronic reminders and simplified medication schedules, have been shown to significantly improve patient adherence [156]. Home health monitoring and education programs have shown significant results in reducing emergency room visits and improving patient adherence and satisfaction [157,158].

Future directions

The management of choledocholithiasis and acute cholangitis in elderly individuals is poised for significant advancements through technological innovation, targeted research, and quality improvement initiatives. Emerging technologies such as AI-driven diagnostic tools, augmented reality for surgical planning, and smart monitoring systems promise to enhance precision and early complication detection, thereby improving patient outcomes [1,159].

Research priorities should focus on developing age-specific treatment algorithms that incorporate geriatric factors, advancing biomarker discovery for early detection, and exploring personalized medicine approaches to optimize treatment efficacy. These efforts are expected to refine the predictive accuracy and increase therapeutic success rates [160,161].

Quality improvement initiatives, including the standardization of care protocols and the integration of telemedicine, are essential for reducing practice variation and improving access to specialized care [162]. By implementing enhanced recovery pathways and leveraging machine learning for risk prediction, healthcare providers can significantly reduce hospital stays and readmission rates [163].

## Conclusions

The management of choledocholithiasis and acute cholangitis in elderly patients demands a comprehensive, evidence-based approach. This review highlights significant advancements across multiple domains: diagnostic capabilities have been enhanced through modern imaging techniques and AI-assisted systems; treatment strategies have evolved toward minimally invasive interventions with personalized approaches; and care delivery has been optimized through enhanced recovery protocols and comprehensive geriatric assessments. The implementation of multidisciplinary approaches and standardized care protocols has shown superior outcomes in this vulnerable population.

As the global population continues to age, the optimization of care for elderly patients with biliary tract diseases becomes increasingly crucial. Future success in managing these conditions will depend on continued technological innovation, the development of age-specific treatment algorithms, and an enhanced focus on prevention and quality-of-life outcomes. Ongoing research and standardization of care protocols remain essential for improving patient outcomes and healthcare delivery in this growing patient population.

## References

[1] Lee TY, Lee SH, Cheon YK, Wang JH (2023). The comparison of clinical outcomes in elderly (≥75 years) and non-elderly (<75 years) patients with acute cholangitis due to choledocholithiasis. Medicina (Kaunas).

[2] Kazi FN, Ghosh S, Sharma JV, Saravanan S, Patil S (2022). Trends in gallbladder disease in young adults: a growing concern. Cureus.

[3] Tan M, Schaffalitzky de Muckadell OB, Laursen SB (2019). Unchanged mortality in patients with acute cholangitis despite an increase in malignant etiologies - a 25-year epidemiological study. Scand J Gastroenterol.

[4] Vos T, Lim SS, Abbafati C (2020). Global burden of 369 diseases and injuries in 204 countries and territories, 1990-2019: a systematic analysis for the Global Burden of Disease Study 2019. Lancet.

[5] Miura F, Okamoto K, Takada T (2018). Tokyo Guidelines 2018: initial management of acute biliary infection and flowchart for acute cholangitis. J Hepatobiliary Pancreat Sci.

[6] Buxbaum JL, Abbas Fehmi SM, Sultan S (2019). ASGE guideline on the role of endoscopy in the evaluation and management of choledocholithiasis. Gastrointest Endosc.

[7] Ramser B, Coleoglou Centeno A, Ferre A, Thomas S, Brooke M, Pieracci F, Morton A (2024). Laparoscopic common bile duct exploration is an effective, safe, and less-costly method of treating choledocholithiasis. Surg Endosc.

[8] Zhang J, Li L, Jiang Y, Li W, Li L (2023). Comparative analysis of laparoscopic choledocholithiasis and ERCP treatment after cholecystectomy. BMC Surg.

[9] Sha Y, Wang Z, Tang R, Wang K, Xu C, Chen G (2024). Modern management of common bile duct stones: breakthroughs, challenges, and future perspectives. Cureus.

[10] Acehan F, Çamlı H, Kalkan C, Tez M, Altiparmak E, Ates I (2023). Characteristics and clinical outcomes of acute cholangitis in older patients. Eur Geriatr Med.

[11] Lammert F, Gurusamy K, Ko CW (2016). Gallstones. Nat Rev Dis Primers.

[12] He J, Nishida S, Xu M, Makishima M, Xie W (2011). PXR prevents cholesterol gallstone disease by regulating biosynthesis and transport of bile salts. Gastroenterology.

[13] Zhou Q, Hu H, Zhao G, Liu P, Wang Y, Zhang H (2021). Effect and related mechanism of Yinchenhao decoction on mice with lithogenic diet-induced cholelithiasis. Exp Ther Med.

[14] Kubica K, Balbus J (2021). A computer study of the risk of cholesterol gallstone associated with obesity and normal weight. Sci Rep.

[15] Smith ZL, Shah R, Elmunzer BJ, Chak A (2022). The next EPISOD: trends in utilization of endoscopic sphincterotomy for sphincter of Oddi dysfunction from 2010-2019. Clin Gastroenterol Hepatol.

[16] Micucci M, Ioan P, Aldini R (2014). Castanea sativa Mill. extract contracts gallbladder and relaxes sphincter of Oddi in guinea pig: a natural approach to biliary tract motility disorders. J Med Food.

[17] Nunes VS, da Silva Ferreira G, Quintão EC (2022). Cholesterol metabolism in aging simultaneously altered in liver and nervous system. Aging (Albany NY).

[18] Lodha M, Chauhan AS, Puranik A (2022). Clinical profile and evaluation of outcomes of symptomatic gallstone disease in the senior citizen population. Cureus.

[19] Rahman SH, Larvin M, McMahon MJ, Thompson D (2005). Clinical presentation and delayed treatment of cholangitis in older people. Dig Dis Sci.

[20] Reynish EL, Hapca SM, De Souza N, Cvoro V, Donnan PT, Guthrie B (2017). Epidemiology and outcomes of people with dementia, delirium, and unspecified cognitive impairment in the general hospital: prospective cohort study of 10,014 admissions. BMC Med.

[21] Agarwal N, Sharma BC, Sarin SK (2006). Endoscopic management of acute cholangitis in elderly patients. World J Gastroenterol.

[22] van Geloven AA, Biesheuvel TH, Luitse JS, Hoitsma HF, Obertop H (2000). Hospital admissions of patients aged over 80 with acute abdominal complaints. Eur J Surg.

[23] Lee S, Chung CW, Ko KH, Kwon SW (2011). Risk factors for the clinical course of cholecystitis in patients who undergo cholecystectomy. Korean J Hepatobiliary Pancreat Surg.

[24] Cagir Y, Durak MB, Simsek C, Yuksel I (2024). Comparison of ERCP outcomes and complication risk between elderly and younger patients: a large single-center study. J Clin Med.

[25] Kaminska H, Szarpak L, Kosior D (2021). Impact of diabetes mellitus on in-hospital mortality in adult patients with COVID-19: a systematic review and meta-analysis. Acta Diabetol.

[26] Popejoy MW, Long J, Huntington JA (2017). Analysis of patients with diabetes and complicated intra-abdominal infection or complicated urinary tract infection in phase 3 trials of ceftolozane/tazobactam. BMC Infect Dis.

[27] Karlawish J (2022). Importance of asking older adults whether they are having difficulty managing finances. JAMA Netw Open.

[28] Abidi S (2017). A knowledge-modeling approach to integrate multiple clinical practice guidelines to provide evidence-based clinical decision support for managing comorbid conditions. J Med Syst.

[29] Mohan R, Wei Lynn Goh S, Tan GW (2021). Validation of Tokyo guidelines 2007 and Tokyo guidelines 2013/2018 criteria for acute cholangitis and predictors of in-hospital mortality. Visc Med.

[30] Goodacre S, Thomas B, Sutton L (2021). Derivation and validation of a clinical severity score for acutely ill adults with suspected COVID-19: The PRIEST observational cohort study. PLoS One.

[31] Thuluvath AJ, Ahn JC, Rattan P (2021). Evaluation of Charcot Triad, Reynolds Pentad, and Tokyo guidelines for diagnosis of cholangitis secondary to choledocholithiasis across patient age groups. Mayo Clin Proc Innov Qual Outcomes.

[32] Makmun D, Fauzi A, Shatri H (2017). Sensitivity and specificity of magnetic resonance cholangiopancreatography versus endoscopic ultrasonography against endoscopic retrograde cholangiopancreatography in diagnosing choledocholithiasis: the Indonesian experience. Clin Endosc.

[33] Isram J, Haider E, Khan RS (2023). Diagnostic accuracy of magnetic resonance cholangiopancreatography in comparison with endoscopic retrograde cholangiopancreatography for detection of the etiology of obstructive jaundice. Cureus.

[34] Suzuki M, Sekino Y, Hosono K (2022). Endoscopic ultrasound versus magnetic resonance cholangiopancreatography for the diagnosis of computed tomography-negative common bile duct stone: Prospective randomized controlled trial. Dig Endosc.

[35] Yeniçeri Ö, Çullu N, Özşeker B, Yeniçeri EN (2019). The accuracy of 3T magnetic resonance cholangiopancreatography in suspected choledocholithiasis. Pol J Radiol.

[36] Meeralam Y, Al-Shammari K, Yaghoobi M (2017). Diagnostic accuracy of EUS compared with MRCP in detecting choledocholithiasis: a meta-analysis of diagnostic test accuracy in head-to-head studies. Gastrointest Endosc.

[37] Tajamul RW, Muteeb SR (2023). Revolutionizing radiology: Exploring applications and advancements in AI for imaging diagnostics. Int Multidiscip Res J.

[38] Ahmed M, Kanotra R, Savani GT (2017). Utilization trends in inpatient endoscopic retrograde cholangiopancreatography (ERCP): a cross-sectional US experience. Endosc Int Open.

[39] Sheng X, Li T, Hu Y, Xiong CS, Hu L (2023). Correlation Between Blood Glucose Indexes Generated by the Flash Glucose Monitoring System and Diabetic Vascular Complications. Diabetes Metab Syndr Obes.

[40] Silangcruz K, Nishimura Y, Czech T, Kimura N, Yess J (2022). Procalcitonin to predict severity of acute cholangitis and need for urgent biliary decompression: systematic scoping review. J Clin Med.

[41] Donkin R, Fung YL, Singh I (2023). Fibrinogen, coagulation, and ageing. Subcell Biochem.

[42] Le Blanc J, Lordkipanidzé M (2019). Platelet function in aging. Front Cardiovasc Med.

[43] Xia F, Hao W, Liang J (2021). Applicability of creatinine-based equations for estimating glomerular filtration rate in elderly Chinese patients. BMC Geriatr.

[44] Wu ZY, Chi SW, Ouyang LJ (2024). Continuous age- and sex-specific reference ranges of liver enzymes in Chinese children and application in pediatric non-alcoholic fatty liver disease. World J Pediatr.

[45] Yang CC, Chen PC, Hsu CW, Chang SL, Lee CC (2015). Validity of the age-adjusted Charlson comorbidity index on clinical outcomes for patients with nasopharyngeal cancer post radiation treatment: a 5-year nationwide cohort study. PLoS One.

[46] Huang J, Ge H, Zhu X, Xue C, Su Q, Chen X, Cheng B (2023). Risk factors analysis and nomogram construction for postoperative pulmonary infection in elderly patients with hip fractures. Aging Clin Exp Res.

[47] Chew J, Chia JQ, Kyaw KK, Fu KJ, Lim C, Chua S, Tan HN (2023). Frailty screening and detection of geriatric syndromes in acute inpatient care: impact on hospital length of stay and 30-day readmissions. Ann Geriatr Med Res.

[48] Park B, Alani Z, Sulistio E (2024). Frailty using the Clinical Frailty Scale to predict short- and long-term adverse outcomes following emergency laparotomy: meta-analysis. BJS Open.

[49] Miłkowska-Dymanowska J, Białas AJ, Szewczyk K, Kurmanowska Z, Górski P, Piotrowski WJ (2018). The usefulness of soluble receptor for advanced glycation end-products in the identification of COPD frequent exacerbator phenotype. Int J Chron Obstruct Pulmon Dis.

[50] Alhammadi E, Kuhlmann JM, Rana M, Frohnhofen H, Moellmann HL (2024). Comprehensive geriatric assessment for predicting postoperative delirium in oral and maxillofacial surgery: a prospective cohort study. Sci Rep.

[51] Buxbaum JL, Buitrago C, Lee A (2021). ASGE guideline on the management of cholangitis. Gastrointest Endosc.

[52] Condon FJ (2019). Choledocholithiasis and Cholangitis: Incidence, Initial Management, and Surgical Management. Multidisciplinary Approaches to Common Surgical Problems.

[53] Khamaysi I, Taha R (2020). ERCP for severe acute cholangitis: the earlier, the better. Turk J Gastroenterol.

[54] Virág M, Leiner T, Rottler M, Ocskay K, Molnar Z (2021). Individualized hemodynamic management in sepsis. J Pers Med.

[55] Goel S, Deshpande SV, Jadawala VH, Suneja A, Singh R (2023). A comprehensive review of postoperative analgesics used in orthopedic practice. Cureus.

[56] Sun Z, Liu C, Huang L (2024). The efficacy of preemptive multimodal analgesia in elderly patients undergoing laparoscopic colorectal surgery: a randomized controlled trial. Sci Rep.

[57] Wang HL, Shao J, Liu WL, Wu F, Chen HB, Zheng RQ, Chen QH (2021). Initial fluid resuscitation (30 mL/kg) in patients with septic shock: more or less?. Am J Emerg Med.

[58] Levy MM, Rhodes A, Phillips GS (2015). Surviving Sepsis Campaign: association between performance metrics and outcomes in a 7.5-year study. Crit Care Med.

[59] Tan TL, Tang YJ, Ching LJ, Abdullah N, Neoh HM (2018). Comparison of Prognostic Accuracy of the quick Sepsis-related Organ Failure Assessment between short- & long-term mortality in Patients Presenting Outside of the intensive care unit - a systematic review & meta-analysis. Sci Rep.

[60] Ohland PL, Jack T, Mast M, Melk A, Bleich A, Talbot SR (2024). Continuous monitoring of physiological data using the patient vital status fusion score in septic critical care patients. Sci Rep.

[61] Soraci L, Cherubini A, Paoletti L (2023). Safety and tolerability of antimicrobial agents in the older patient. Drugs Aging.

[62] Strich JR, Heil EL, Masur H (2020). Considerations for empiric antimicrobial therapy in sepsis and septic shock in an era of antimicrobial resistance. J Infect Dis.

[63] Maillard A, Delory T, Bernier J (2023). Effectiveness of third-generation cephalosporins or piperacillin compared with cefepime or carbapenems for severe infections caused by wild-type AmpC β-lactamase-producing Enterobacterales: a multi-centre retrospective propensity-weighted study. Int J Antimicrob Agents.

[64] Herrmann L, Kimmig A, Rödel J, Hagel S, Rose N, Pletz MW, Bahrs C (2021). Early treatment outcomes for bloodstream infections caused by potential AMPC beta-lactamase-producing Enterobacterales with focus on piperacillin/tazobactam: a retrospective cohort study. Antibiotics (Basel).

[65] Cheng MP, Lee RS, Cheng AP (2019). Beta-lactam/beta-lactamase inhibitor therapy for potential AMPC-producing organisms: a systematic review and meta-analysis. Open Forum Infect Dis.

[66] Paterson IK, Hoyle A, Ochoa G, Baker-Austin C, Taylor NG (2016). Optimising antibiotic usage to treat bacterial infections. Sci Rep.

[67] Hochreiter M, Köhler T, Schweiger AM, Keck FS, Bein B, von Spiegel T, Schroeder S (2009). Procalcitonin to guide duration of antibiotic therapy in intensive care patients: a randomized prospective controlled trial. Crit Care.

[68] Al-Maqbali JS, Shukri ZA, Sabahi NA, Al-Riyami I, Al Alawi AM (2022). Vancomycin therapeutic drug monitoring (TDM) and its association with clinical outcomes: a retrospective cohort. J Infect Public Health.

[69] Ak O, Diktas H, Senbayrak S, Saltoglu N (2020). Skin and soft tissue infections: diagnosis and therapy. Klimik Derg.

[70] de Jong E, van Oers JA, Beishuizen A (2016). Efficacy and safety of procalcitonin guidance in reducing the duration of antibiotic treatment in critically ill patients: a randomised, controlled, open-label trial. Lancet Infect Dis.

[71] Tarikci Kilic E, Kahraman R, Ozdil K (2019). Evaluation of safety and outcomes of endoscopic retrograde cholangiopancreatography in 1337 patients at a single center. Medeni Med J.

[72] Anderson MA, Fisher L, Jain R (2012). Complications of ERCP. Gastrointest Endosc.

[73] Testoni PA, Mariani A, Giussani A (2010). Risk factors for post-ERCP pancreatitis in high- and low-volume centers and among expert and non-expert operators: a prospective multicenter study. Am J Gastroenterol.

[74] Ergin E, Oruç N, Ersöz G, Tekeşin O, Özütemiz Ö (2021). Prognosis and risk factors of ERCP pancreatitis in elderly. Sci Rep.

[75] Park N, Lee SH, You MS (2021). Optimal timing of endoscopic retrograde cholangiopancreatography for acute cholangitis associated with distal malignant biliary obstruction. BMC Gastroenterol.

[76] Kim H, Shin SP, Hwang JW, Lee JW (2020). Outcomes of laparoscopic common bile duct exploration (LCBDE) after failed endoscopic retrograde cholangiopancreatography versus primary LCBDE for managing cholecystocholedocholithiasis. J Int Med Res.

[77] Manivasagam SS, Chandra JN, Shah S, Kuraria V, Manocha P (2024). Single-stage laparoscopic common bile duct exploration and cholecystectomy versus two-stage endoscopic stone extraction followed by laparoscopic cholecystectomy for patients with cholelithiasis and choledocholithiasis: a systematic review. Cureus.

[78] Zhang HW, Chen YJ, Wu CH, Li WD (2014). Comparison between laparoscopic and conventional technique in the surgical treatment of choledocholithiasis. Am Surg.

[79] Chandrashekhara SH, Gamanagatti S, Singh A, Bhatnagar S (2016). Current status of percutaneous transhepatic biliary drainage in palliation of malignant obstructive jaundice: a review. Indian J Palliat Care.

[80] Yang J, Liu Y, Liu S (2023). Timing of biliary decompression for acute cholangitis. World J Gastroenterol.

[81] Nirhali R, Bhoir R, Prajapati R (2024). Systematic review of percutaneous cholecystostomy (PC) as definitive vs bridge therapy for acute cholecystitis in high-risk patients. Indian J Surg.

[82] Partridge JS, Moonesinghe SR, Lees N, Dhesi JK (2022). Perioperative care for older people. Age Ageing.

[83] Choi JU, Kee TH, Lee DH, Hwang CJ, Park S, Cho JH (2024). Enhanced recovery after surgery protocols in one- or two-level posterior lumbar fusion: improving postoperative outcomes. J Clin Med.

[84] Guazzi M, Arena R, Halle M, Piepoli MF, Myers J, Lavie CJ (2018). 2016 focused update: clinical recommendations for cardiopulmonary exercise testing data assessment in specific patient populations. Eur Heart J.

[85] Nadruz W Jr, West E, Sengeløv M (2017). Prognostic Value of Cardiopulmonary Exercise Testing in Heart Failure With Reduced, Midrange, and Preserved Ejection Fraction. J Am Heart Assoc.

[86] Yin K, Ding J, Wu Y, Peng M (2018). Goal-directed fluid therapy based on noninvasive cardiac output monitor reduces postoperative complications in elderly patients after gastrointestinal surgery: a randomized controlled trial. Pak J Med Sci.

[87] Federico WA, De Moraes CM, De Carvalho R (2024). Lesões por pressão decorrentes do posicionamento cirúrgico: ocorrência e fatores de risco. Revista SOBECC.

[88] Tartari E, Weterings V, Gastmeier P, Rodríguez Baño J, Widmer A, Kluytmans J, Voss A (2017). Patient engagement with surgical site infection prevention: an expert panel perspective. Antimicrob Resist Infect Control.

[89] Cruz LD, Danieli F, Håkansson MÅ, Johansson ML, Dos Santos FR, Mirândola Barbosa Reis AC, Hyppolito MA (2023). Minimally invasive surgery as a new clinical standard for bone anchored hearing implants-real-world data from 10 years of follow-up and 228 surgeries. Front Surg.

[90] Tayar DO, Ribeiro U Jr, Cecconello I, Magalhães TM, Simões CM, Auler JO Jr (2018). Propensity score matching comparison of laparoscopic versus open surgery for rectal cancer in a middle-income country: short-term outcomes and cost analysis. Clinicoecon Outcomes Res.

[91] Zhu W, Yan Y, Sun Y (2021). Implementation of Enhanced Recovery After Surgery (ERAS) protocol for elderly patients receiving surgery for intertrochanteric fracture: a propensity score-matched analysis. J Orthop Surg Res.

[92] Leo L, Baisi A, Raveglia F (2023). Effetti della mobilizzazione precoce sull'incidenza di complicanze postoperatorie nei pazienti sottoposti a toracoscopia: uno studio randomizzato controllato. Dissertation Nursing.

[93] Alqaisi OM, Al-Ghabeesh S (2024). Quality of postoperative pain management in orthopedic patients and its impact on sleep quality and patient satisfaction: an integrative review. Cureus.

[94] Vadeghani AT, Grant M, Forget P (2024). Perioperative pain management interventions in opioid user patients: an overview of reviews. BMC Anesthesiol.

[95] Cambise C, De Cicco R, Luca E (2024). Postoperative urinary retention (POUR): a narrative review. Saudi J Anaesth.

[96] Zhang H, Lu Y, Liu M, Zou Z, Wang L, Xu FY, Shi XY (2013). Strategies for prevention of postoperative delirium: a systematic review and meta-analysis of randomized trials. Crit Care.

[97] Jackson J, Davies P, Leggett N (2019). Systematic review of interventions for the prevention and treatment of postoperative urinary retention. BJS Open.

[98] Tsarouchi MI, Hoxhaj A, Mann RM (2023). New approaches and recommendations for risk-adapted breast cancer screening. J Magn Reson Imaging.

[99] Hull MA, Rees CJ, Sharp L, Koo S (2020). A risk-stratified approach to colorectal cancer prevention and diagnosis. Nat Rev Gastroenterol Hepatol.

[100] Li Y, Jia K, Jia Y, Yang Y, Yao Y, Chen M, Peng Y (2021). Understanding the predictive value and methods of risk assessment based on coronary computed tomographic angiography in populations with coronary artery disease: a review. Precis Clin Med.

[101] Hornor MA, Ma M, Zhou L, Cohen ME, Rosenthal RA, Russell MM, Ko CY (2020). Enhancing the American College of Surgeons NSQIP surgical risk calculator to predict geriatric outcomes. J Am Coll Surg.

[102] Mohan R, Goh SW, Tan G, Junnarkar SP, Huey C, Shelat VG (2021). Validation of TG07 and TG13/TG18 criteria for acute cholangitis and predictors of in-hospital mortality in patients over 80 years old. Clin Exp Hepatol.

[103] Sato Y, Kaneko R, Yano Y (2022). Volume-outcome relationship in cancer survival rates: analysis of a regional population-based cancer Registry in Japan. Healthcare (Basel).

[104] Seo YJ, Sareh S, Hadaya J, Sanaiha Y, Ziaeian B, Shemin RJ, Benharash P (2022). Factors associated with high resource use in elective adult cardiac surgery from 2005 to 2016. Ann Thorac Surg.

[105] Wei Y, Ming J, Shi L, Ke X, Sun H, Chen Y (2020). Physician-patient shared decision making, patient satisfaction, and adoption of new health technology in China. Int J Technol Assess Health Care.

[106] Amir AH, Davey MG, Donlon NE (2024). Evaluating the impact of enhanced recovery after surgery protocols following emergency laparotomy - a systematic review and meta-analysis of randomised clinical trials. Am J Surg.

[107] Nelson LD, Temkin NR, Barber J (2023). Functional recovery, symptoms, and quality of life 1 to 5 years after traumatic brain injury. JAMA Netw Open.

[108] Liang W, Qin G, Yu L, Wang Y (2023). Reducing complications of femoral neck fracture management: a retrospective study on the application of multidisciplinary team. BMC Musculoskelet Disord.

[109] Prusaczyk B, Burke RE (2023). It's time for the field of geriatrics to invest in implementation science. BMJ Qual Saf.

[110] Sarkar K (2018). Heart Team-the Indian perspective. Indian J Thorac Cardiovasc Surg.

[111] Zaij S, Pereira Maia K, Leguelinel-Blache G, Roux-Marson C, Kinowski JM, Richard H (2023). Intervention of pharmacist included in multidisciplinary team to reduce adverse drug event: a qualitative systematic review. BMC Health Serv Res.

[112] Zhang L, Ren XY, Huang HX (2022). Development of the practice of pharmaceutical care for cancer pain management in outpatient clinics using the Delphi method. Front Pharmacol.

[113] Gómez-Izquierdo JC, Trainito A, Mirzakandov D (2017). Goal-directed fluid therapy does not reduce primary postoperative ileus after elective laparoscopic colorectal surgery: a randomized controlled trial. Anesthesiology.

[114] Kapritsou M, Alexandrou E, Konstantinou EA, Giannakopoulou M, Fyrfiris N, Korkolis DP (2022). Clinical outcomes of enhanced recovery after surgery protocol for hepato-pancreato-biliary surgery; a five-year experience from a Hellenic oncological hospital eras protocol and HPB surgery. Health Res J.

[115] Tura I, Erden S (2023). Examination of pain assessment and multimodal analgesia records in trauma patients. Online Türk Sağlık Bilimleri Dergisi.

[116] Lau BD, Murphy P, Nastasi AJ (2020). Effectiveness of ambulation to prevent venous thromboembolism in patients admitted to hospital: a systematic review. CMAJ Open.

[117] Clark DE, Lowman JD, Griffin RL, Matthews HM, Reiff DA (2013). Effectiveness of an early mobilization protocol in a trauma and burns intensive care unit: a retrospective cohort study. Phys Ther.

[118] Mcmartin K (2013). Discharge planning in chronic conditions: an evidence-based analysis. Ont Health Technol Assess Ser.

[119] Liu K, Ogura T, Takahashi K (2019). A progressive early mobilization program is significantly associated with clinical and economic improvement: a single-center quality comparison study. Crit Care Med.

[120] Rodriguez-Pascual C, Paredes-Galan E, Vilches-Moraga A, Ferrero-Martinez AI, Torrente-Carballido M, Rodriguez-Artalejo F (2014). Comprehensive geriatric assessment and 2-year mortality in elderly patients hospitalized for heart failure. Circ Cardiovasc Qual Outcomes.

[121] Wang MC, Liao WC, Lee KC, Lu SH, Lin YP (2022). Validation of screening tools for predicting the risk of functional decline in hospitalized elderly patients. Int J Environ Res Public Health.

[122] Hutchings L, Fox R, Chesser T (2011). Proximal femoral fractures in the elderly: how are we measuring outcome?. Injury.

[123] Tan TC, Guo YY, Ho DJ (2023). Reference values, determinants and regression equation for the Timed-Up and Go test (TUG) in healthy Asian population aged 21 to 85 years. Int J Environ Res Public Health.

[124] Khera T, Helfand J, Kelly L, Mueller A, Shankar P, Marcantonio ER, Subramaniam B (2022). Twelve-month cognitive and functional outcomes following cardiac surgery: the DEXACET trial of intravenous acetaminophen versus placebo. Front Pharmacol.

[125] Danquah MO, Yan E, Lee JW (2024). The utility of the Montreal cognitive assessment (MoCA) in detecting cognitive impairment in surgical populations - a systematic review and meta-analysis. J Clin Anesth.

[126] Mostafa OE, Al-Allaf O, Tahir M, Hossain F, Blackwell J (2024). Do hypoalbuminaemia increase the risk of surgical site infection in neck of femur fracture patients: a systematic review and meta-analysis. Cureus.

[127] Yam CH, Wong EL, Cheung AW, Chan FW, Wong FY, Yeoh EK (2012). Framework and components for effective discharge planning system: a Delphi methodology. BMC Health Serv Res.

[128] Vasilevskis EE, Shah AS, Hollingsworth EK, Shotwell MS, Kripalani S, Mixon AS, Simmons SF (2023). Deprescribing medications among older adults from end of hospitalization through postacute care: a shed-MEDS randomized clinical trial. JAMA Intern Med.

[129] Cai Q, Grigoroglou C, Allen T, Chen TC, Chen LC, Kontopantelis E (2024). Interventions to reduce opioid use for patients with chronic non-cancer pain in primary care settings: a systematic review and meta-analysis. PLoS One.

[130] Al-Alawi KS, Al-Azri M, Al-Fahdi A, Chan MF (2022). Effect of psycho-educational intervention to reduce anxiety and depression at postintervention and follow-up in women with breast cancer: a systematic review and meta-analysis. Semin Oncol Nurs.

[131] Rasmus P, Lipert A, Pękala K (2021). The influence of a psychosocial rehabilitation program in a community health setting for patients with chronic mental disorders. Int J Environ Res Public Health.

[132] Kellezi B, Wakefield JR, Stevenson C (2019). The social cure of social prescribing: a mixed-methods study on the benefits of social connectedness on quality and effectiveness of care provision. BMJ Open.

[133] Sanjuán M, Navarro E, Calero MD (2023). Caregiver training: evidence of its effectiveness for cognitive and functional improvement in older adults. J Clin Nurs.

[134] Burgdorf JG, Arbaje AI, Chase JA, Wolff JL (2022). Current practices of family caregiver training during home health care: a qualitative study. J Am Geriatr Soc.

[135] Beidelschies M, Alejandro-Rodriguez M, Ji X, Lapin B, Hanaway P, Rothberg MB (2019). Association of the functional medicine model of care with patient-reported health-related quality-of-life outcomes. JAMA Netw Open.

[136] Wang Y, Lu J, Wen N (2022). The role of diet and nutrition related indicators in biliary diseases: an umbrella review of systematic review and meta-analysis. Nutr Metab (Lond).

[137] Bhojani N, Bjazevic J, Wallace B (2022). UPDATE - Canadian Urological Association guideline: evaluation and medical management of kidney stones. Can Urol Assoc J.

[138] Cheungpasitporn W, Rossetti S, Friend K, Erickson SB, Lieske JC (2016). Treatment effect, adherence, and safety of high fluid intake for the prevention of incident and recurrent kidney stones: a systematic review and meta-analysis. J Nephrol.

[139] Wang X, Xu X, Wu J, Zhu Y, Lin Y, Zheng X, Xie L (2015). Systematic review and meta-analysis of the effect of alcohol intake on the risk of urolithiasis including dose-response relationship. Urol Int.

[140] Shanmugam H, Molina Molina E, Di Palo DM (2020). Physical activity modulating lipid metabolism in gallbladder diseases. J Gastrointestin Liver Dis.

[141] Mills E, Eyawo O, Lockhart I, Kelly S, Wu P, Ebbert JO (2011). Smoking cessation reduces postoperative complications: a systematic review and meta-analysis. Am J Med.

[142] Ruffin L (2017). Health coaching strategy to improve glycemic control in African- American adults with type 2 diabetes: an integrative review. J Natl Black Nurses Assoc.

[143] Sharahili AY, Mir SA, ALosari S (2023). Correlation of HbA1c level with lipid profile in type 2 diabetes mellitus patients visiting a primary healthcare center in Jeddah City, Saudi Arabia: a retrospective cross-sectional study. Diseases.

[144] Singh R, Zogg H, Ghoshal UC, Ro S (2022). Current treatment options and therapeutic insights for gastrointestinal dysmotility and functional gastrointestinal disorders. Front Pharmacol.

[145] Cieslak KP, Baur O, Verheij J, Bennink RJ, van Gulik TM (2016). Liver function declines with increased age. HPB (Oxford).

[146] Al-Shawi MM, Aljama NA, Aljedani R, Alsaleh MH, Atyia N, Alsedrah A, Albardi M (2022). The role of radiological imaging in the diagnosis and treatment of urolithiasis: a narrative review. Cureus.

[147] Chen X, Yan XR, Zhang LP (2018). Ursodeoxycholic acid after common bile duct stones removal for prevention of recurrence: a systematic review and meta-analysis of randomized controlled trials. Medicine (Baltimore).

[148] Arumugam G, Nagarathna R, Majumdar V (2020). Yoga-based lifestyle treatment and composite treatment goals in type 2 diabetes in a rural South Indian setup- a retrospective study. Sci Rep.

[149] Hill KG, Woodward D, Woelfel T, Hawkins JD, Green S (2016). Planning for long-term follow-up: strategies learned from longitudinal studies. Prev Sci.

[150] Aubrey-Bassler K, Fernandes C, Penney C (2019). The effectiveness of a proven chronic disease prevention and screening intervention in diverse and remote primary care settings: an implementation study on the BETTER 2 Program. BJGP Open.

[151] Wu M, Xia L, Zhang L, Xu Y, Cheng Y, Zhang X, Chen L (2023). Perioperative management for elderly patients undergoing day surgery: evidence-based practice for nursing care and day surgery. Front Med (Lausanne).

[152] ElSayed NA, Aleppo G, Aroda VR (2023). 8. Obesity and weight management for the prevention and treatment of type 2 diabetes: standards of care in diabetes-2023. Diabetes Care.

[153] Jiang LG, Zhang Y, Greca E (2022). Emergency department Patient Navigator Program demonstrates reduction in emergency department return visits and increase in follow-up appointment adherence. Am J Emerg Med.

[154] Adams RJ (2010). Improving health outcomes with better patient understanding and education. Risk Manag Healthc Policy.

[155] Wong A, Huang Y, Sowa PM, Banks MD, Bauer JD (2022). Effectiveness of dietary counseling with or without nutrition supplementation in hospitalized patients who are malnourished or at risk of malnutrition: a systematic review and meta-analysis. JPEN J Parenter Enteral Nutr.

[156] Saha SK, Adhikary A, Jha A, Saha S, Bora B (2022). Probability of medication adherence when alarm is used as a reminder. Int J Reliab Qual E-Healthc.

[157] Xyrichis A, Fletcher S, Philippou J, Brearley S, Terblanche M, Rafferty AM (2021). Interventions to promote family member involvement in adult critical care settings: a systematic review. BMJ Open.

[158] Khanal MK, Bhandari P, Dhungana RR (2021). Effectiveness of community-based health education and home support program to reduce blood pressure among patients with uncontrolled hypertension in Nepal: a cluster-randomized trial. PLoS One.

[159] Larrain C, Torres-Hernandez A, Hewitt DB (2024). Artificial intelligence, machine learning, and deep learning in the diagnosis and management of hepatocellular carcinoma. Livers.

[160] Johnson KB, Wei WQ, Weeraratne D (2021). Precision medicine, AI, and the future of personalized health care. Clin Transl Sci.

[161] Chen Y, Deng X, Lin D (2023). Predicting 1-, 3-, 5-, and 8-year all-cause mortality in a community-dwelling older adult cohort: relevance for predictive, preventive, and personalized medicine. EPMA J.

[162] Liu JC, Cheng CY, Cheng TH, Liu CN, Chen JJ, Hao WR (2024). Unveiling the potential: remote monitoring and telemedicine in shaping the future of heart failure management. Life (Basel).

[163] Davis S, Zhang J, Lee I, Rezaei M, Greiner R, McAlister FA, Padwal R (2022). Effective hospital readmission prediction models using machine-learned features. BMC Health Serv Res.

